# Quasi‐Antipolar Nanoclusters Driven Superior Energy Storage in High‐Entropy Relaxor Ferroelectrics

**DOI:** 10.1002/advs.202522801

**Published:** 2025-12-30

**Authors:** Ao Tian, Zehao Li, Qingkang Jiang, Xiang Wu, Xuewen Jiang, Xin Gao, Xiaokuo Er, Mohamed Mahmoud, Maqbool Ur Rehman, Liqiang Liu, Aiwen Xie, Tengfei Hu, Ruzhong Zuo

**Affiliations:** ^1^ Center for Advanced Ceramics School of Materials Science and Engineering Anhui Polytechnic University Wuhu P. R. China; ^2^ The Key Lab of Inorganic Functional Materials and Devices Shanghai Institute of Ceramics Chinese Academy of Sciences Shanghai P. R. China; ^3^ Anhui Key Laboratory of Low Temperature Co‐fired Materials School of Chemistry and Materials Engineering Huainan Normal University Huainan P. R. China

**Keywords:** antiferroelectric, energy storage, in situ structural evolution, multilayer ceramic capacitors, relaxor ferroelectrics

## Abstract

Relaxor ferroelectrics featuring highly dynamic polar nanoregions hold significant potential for pulse‐power dielectric capacitor applications. Nevertheless, achieving an optimal polarization‐field response that combines low hysteresis, delayed polarization saturation, and high maximum polarization remains a critical challenge toward superior comprehensive energy storage performance. Herein, we propose an effective strategy of engineering quasi‐antipolar nanoclusters in relaxor ferroelectrics via a high‐entropy composition design to optimize polarization behavior. By intentionally incorporating aliovalent ions with different ferroelectric activities into antiferroelectric NaNbO_3_, local antiparallel‐like polarization configurations were constructed within a high‐entropy relaxor matrix of Na_0.73_Ba_0.1_Bi_0.11_Li_0.06_Nb_0.73_Ti_0.22_Fe_0.05_O_3_ (NBBLNTF). These quasi‐antipolar nanoclusters not only weaken the coupling among polar nanoregions but also exhibit distinct transition behaviors under high electric fields toward a ferroelectric state. Consequently, a polarization‐field loop with low hysteresis, high linearity, and large maximum polarization is achieved, yielding an ultrahigh recoverable energy density *W_rec_* of 18.3 J·cm^−3^ with a high efficiency *η* of 91.5% and an outstanding energy storage strength *W_rec_/E* of 0.25 J/(kV·mm^−5^) in NBBLNTF multilayer ceramic capacitors, together with excellent thermal and frequency stability. These results offer a feasible strategy for developing next‐generation high‐performance dielectrics with exceptional energy storage properties.

## Graphical abstract

Quasi‐antipolar nanoclusters are engineered in lead‐free NaNbO_3_‐based high‐entropy relaxors that weaken polar nanoregion coupling and induce distinct ferroelectric transition under high fields to enable desirable polarization response. This breakthrough system achieves ultrahigh recoverable‐energy‐density  (≈18.3 J cm^−3^), efficiency (≈91.5%), and breakthrough energy‐storage‐strength *(0.25* J/(kV·mm^−5^)), supporting superior energy‐storage in eco‐friendly multilayer ceramic capacitors.

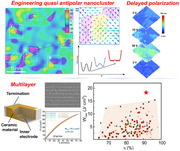

## 1 Introduction

Dielectric energy‐storage capacitors play a vital role in modern electronic and electrical power systems, particularly for pulse‐power applications such as medical defibrillators, hybrid electric vehicles, and advanced weapons systems, where rapid charge/discharge cycles are essential [1, 2, 3, 4]. Among various types of dielectric capacitors, ceramic‐based capacitors, especially multilayer ceramic capacitors (MLCCs), stand out due to their compact size, fast switching speed, excellent thermal stability, and high reliability, making them commercially indispensable [5, 6]. Dielectric ceramics used for energy storage primarily include linear dielectrics, ferroelectrics (FEs), relaxor FEs, and antiferroelectrics (AFEs), each exhibiting distinct polarization‐electric field (*P‐E*) responses that directly govern their energy storage performance [7].

Among these dielectric families, AFEs have attracted considerable interest for energy storage owing to their characteristic field‐induced AFE‐to‐FE phase transition, which yields a large maximum polarization (*P_max_*) and thus a high theoretical energy storage density (*W_rec_*) [8, 9]. However, this first‐order phase transition is invariably accompanied by substantial hysteresis, leading to low energy‐storage efficiency (*η*). In contrast, relaxor FEs typically exhibit slim *P‐E* loops with minimal hysteresis as a result of their highly dynamic polar nanoregions (PNRs), which give rise to high *η* [10, 11, 12]. Nonetheless, their relatively low *P_max_* and premature polarization saturation generally limit the achievable *W_rec_*. Recent research has sought to overcome the trade‐off between *W_rec_* and *η* in relaxor FEs through various strategies, including constructing multi‐phase coexistence, exploiting superparaelectric states, or designing supercritical relaxors [2, 13, 14, 15, 16]. Despite these advances, many studies have overlooked the importance of the energy storage strength (*W_rec_*/*E*), a critical metric defined as the recoverable energy density per unit electric field [17]. A high *W_rec_*/*E* value is essential for achieving superior energy‐storage performance under relatively low applied fields, which in turn enhances device reliability and facilitates miniaturization [18, 19]. Therefore, an ideal energy‐storage feature necessitates the simultaneous optimization of *W_rec_*, *η*, and *W_rec_*/*E*, which fundamentally stems from an optimal *P‐E* response featuring high maximum polarization, low hysteresis, and delayed polarization saturation (as illustrated schematically in Figure S1).

**FIGURE 1:**
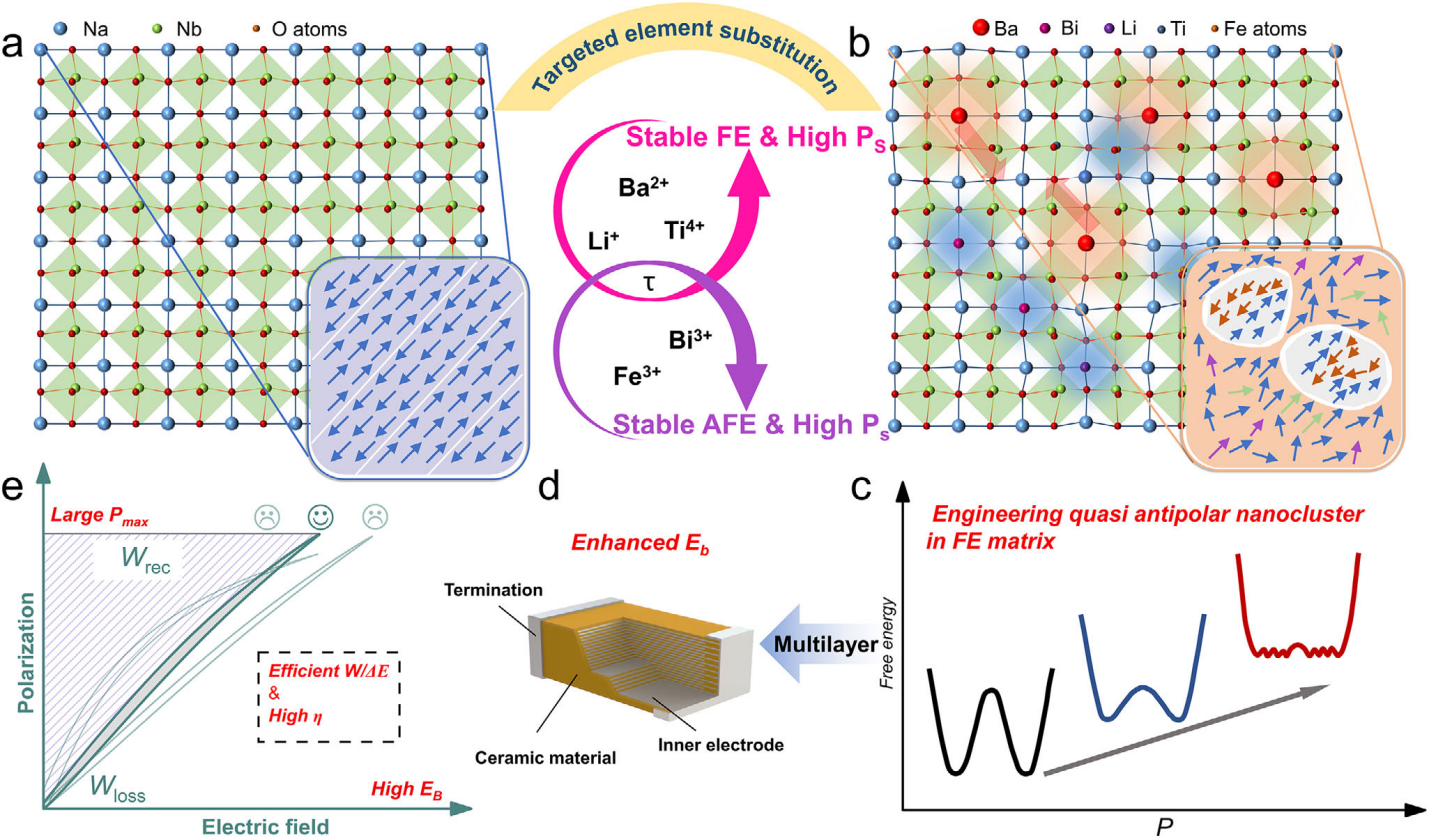
Schematic diagram for engineering QA‐NCs strategy within a high‐entropy relaxor FE matrix for a high‐performance energy storage dielectric capacitor. By incorporating aliovalent ions with different *τ* and high FE activities (spontaneous polarization, *P_s_*), QA‐NCs were constructed in relaxor ferroelectrics, thereby achieving a nonlinear *P‐E* response with large *P_max_* and high breakdown strength *E_B_* in NBBLNTF MLCC.

Recently, several attempts have been made to combine the advantages of AFEs and relaxor FEs by constructing local‐scale coexistence of AFE domains and PNRs [20, 21]. However, the resulting energy‐storage feature often remains a compromise between *W_rec_* and *η*. This limitation arises because harnessing the high polarization of AFEs necessitates an AFE‐to‐FE phase transition, whose intrinsic hysteresis inevitably degrades *η*. Moreover, these systems mostly require extremely high electric fields to achieve promising performance, failing to address the challenge of low *W_rec_*/*E* [22]. In this work, we propose a novel strategy to optimize the *P‐E* response for advanced energy storage by engineering quasi‐antipolar nanoclusters (QA‐NCs) within a high‐entropy relaxor FE matrix, as illustrated in Figure 1. Unlike conventional AFE domains (Figure 1a), these QA‐NCs are composed of PNRs with large polarization orientation differences (>90°), yet not perfectly antiparallel (Figure 1b). This pronounced polarization mismatch promotes local stability, akin to geometric frustration, which effectively weakens the correlations among surrounding PNRs. Furthermore, the electric‐field‐induced transition of QA‐NCs toward a ferroelectric state differs from that of typical PNRs, requiring higher driving fields and thereby contributing to delayed polarization saturation. This results in a special free energy landscape with a flattened, multi‐well profile and low energy barriers for the short‐range ordered PNRs rotation and QA‐NCs to FE transitions (Figure 1c). Therefore, the optimized nonlinear *P‐E* response is realized. Regarding polarization hysteresis, the extremely small PNRs in high‐entropy relaxor FEs exhibit high polarization responsiveness to electric fields, while the energy barrier for the transition of QA‐NCs to a FE state is lower than that of conventional AFE nanodomains, thus avoiding a significant increase in hysteresis. As a result, extremely low polarization hysteresis can be achieved. Additionally, the polymorphic nanodomain structure contributes to a high *P_max_*. To realize this strategy, we conducted a high‐entropy composition design of intentionally incorporating aliovalent ions with different FE activities and tolerance factors (τ) into the A and B sites of AFE/FE‐tunable NaNbO_3_ (NN) matrix. As the configurational entropy increases, the compositional disorder is significantly enhanced. Meanwhile, the local chemical heterogeneity, where some species tend to stabilize FE order while others favor AFE order, is anticipated to facilitate the formation of such quasi‐antipolar configurations. The designed Na_0.73_Ba_0.1_Bi_0.11_Li_0.06_Nb_0.73_Ti_0.22_Fe_0.05_O_3_ (NBBLNTF) high‐entropy (≈1.58R) relaxor ceramic demonstrates a *W_rec_* of 18.3 J·cm^−3^ and *η* of 91.5%, with an efficient *W_rec_*/*ΔE* of 0.25 J/(kV·mm^5^) in its prototype MLCC device, validating the feasibility of the proposed quasi‐antipolar nanocluster engineering strategy.

## 2 Results and Discussion

**FIGURE 2:**
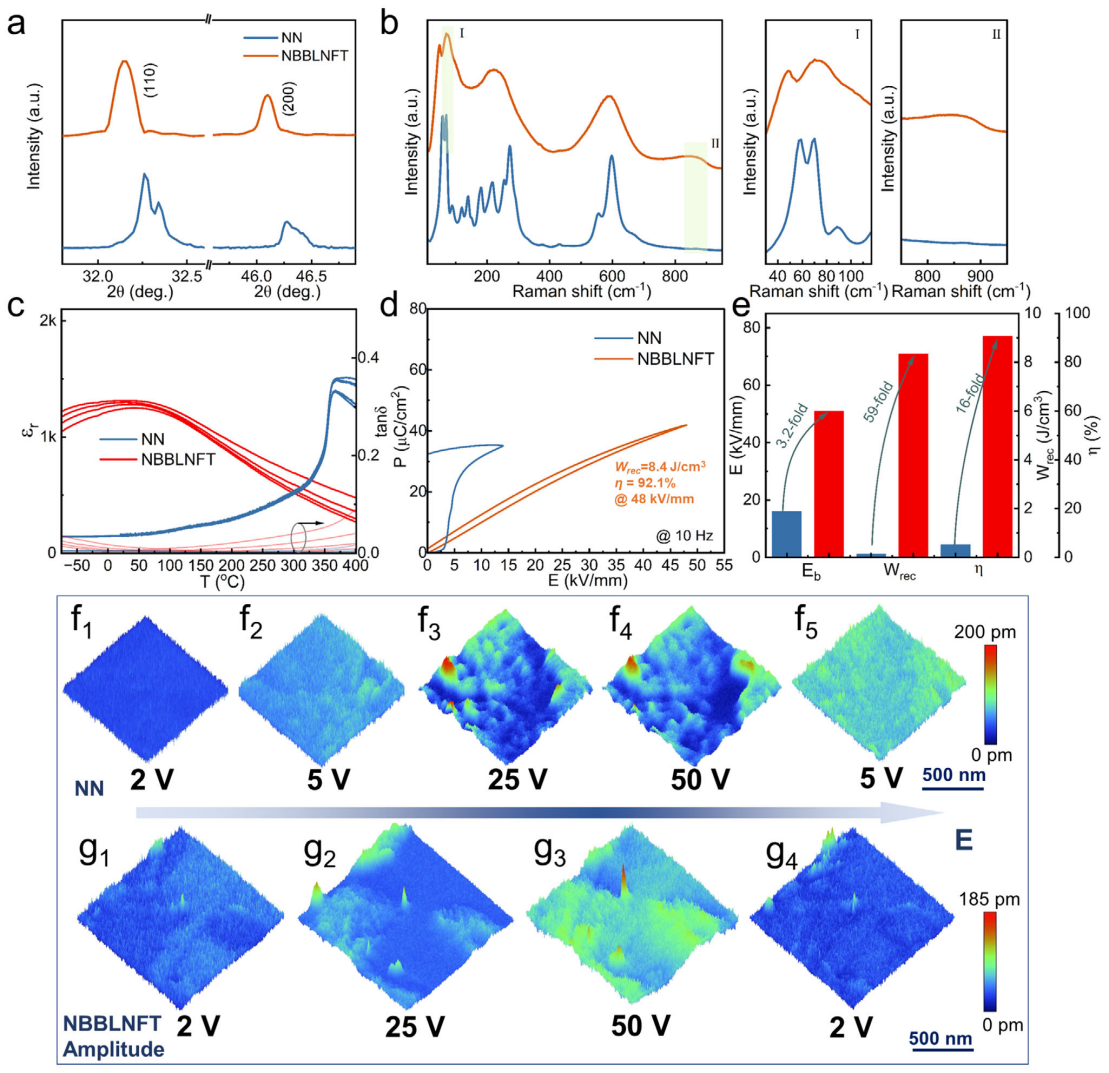
Phase structure, capacitance, microstructure, and piezoelectric response of NN and NBBLNTF ceramics. (a) Enlarged XRD patterns. (b) Raman spectra. (c) Temperature‐dependent dielectric constant *ε_r_* and loss *tanδ*. (d) *P‐E* loops at the maximum applied electric field with a test frequency of 10 Hz. (e) Comparison of the breakdown strength *E_b_*, energy density *W_rec_*, and efficiency *η*. Out‐of‐plane PFM amplitude images at various electric fields of (f) NN, and (g) NBBLNTF ceramic.

The X‐ray diffraction (XRD) pattern (Figure S2a) confirms the pure perovskite structure of the NBBLNTF ceramic. The NN ceramic exhibits an antiferroelectric orthorhombic phase with two distinct splitting peaks (002)_O_/(200)_O_ and the presence of (11 3/4) and (21 3/4) superlattice diffraction peaks (Figure 2a; Figure S2b). Following multielement substitution, the NBBLNTF ceramic demonstrates a pseudocubic average structure as confirmed by the split diffraction lines merging, which is typical in relaxor materials. The local structure changes are analyzed by Raman spectroscopy in Figure 2b. Distinct multi‐peak features below 300 cm^−1^, characteristic of the AFE orthorhombic P phase, can be observed in pure NN [23]. NBBLNTF ceramic shows the obvious broadened Raman peaks, indicating increased structural disorder attributed to the break of the long‐range ordering by the local chemical heterogeneity in the high entropy ceramic [24, 25]. Figure 2c presents the temperature‐dependent dielectric spectra collected during the heating process. Pure NN ceramic obtains a maximum permittivity (*ε_m_*) near 370 °C with a sharp dielectric anomaly. After the configurational entropy modulation of aliovalent ions incorporated, the high entropy NBBLNTF ceramic with an entropy value of 1.58R exhibits a significantly enhanced relaxor behavior with the flattened permittivity‐temperature response, as indicated by the enhanced relaxation degree (*ΔT_relaxor_* = *T_m,1 MHz_*−*T_m,1 kHz_*) and diffuseness degree (*γ*) (Figure S3). A large relaxor degree would contribute to the exceptional energy storage performance, as the presence of high‐dynamic PNRs exhibits a rapid polarization response to an external electric field, resulting in an improvement in efficiency. Additionally, the dielectric properties demonstrate excellent temperature stability, with the capacitance variation rate (*ΔC/C*) remaining within ±15% across a broad temperature range from −100°C to 157°C (Figure S4), which is beneficial for the stability of energy storage performance. Figure 2d displays the polarization versus electric field (*P*‐*E*) hysteresis loops of NN and NBBLNTF ceramic measured from low electric field to breakdown using a bipolar triangular external field at 10 Hz. Consistent with the significantly enhanced relaxation degree, a greatly slimmed loop with significantly reduced hysteresis loss, i.e., from typical FE features of NN ceramic with a large *P_r_* ≈32 µC·cm^−2^ to a near‐zero *P_r_* ≈0.43 µC·cm^−2^ is shown in NBBLNTF ceramic. Meanwhile, the tested electric field is greatly enhanced in NBBLNTF ceramic. The Weibull distribution fitting reveals that the statistical breakdown strength *E_b_* values increase from 16 kV·mm^−1^ in pure NN to 51 kV·mm^−1^ in NBBLNTF ceramic. *E_b_* serves as a critical parameter determining dielectric energy storage performance. This improvement stems from the superior electrical insulation, characterized by increased resistance and higher activation energy *E_a_* due to grain refinement, which effectively suppresses the leakage current. (Figure S5). Consequently, a substantial enhancement in energy storage performance with *W_rec_* of 8.8 J cm^−3^ and *η* of 91.7% (nearly 59‐fold in *W_rec_*, and 16‐fold in *η*) is achieved in NBBLNTF ceramic, benefiting from the markedly elevated E_b_ value and optimized polarization behavior, as shown in Figure 2e.

The domain response to the applied electric fields after multielement substitution is detected utilizing in situ piezoresponse force microscopy (PFM). The polarization behavior can be evaluated by the variation of amplitude magnitude under different electric fields, as presented in Figure 2f,g. The NN ceramic exhibits amplitude responsiveness under a driving electric field (≈5 V), whereas pronounced responsiveness can be observed under a low driving electric field (≈2 V) in NBBLNTF ceramic, demonstrating the dominance of highly dynamic PNRs in NBBLNTF ceramic. With increasing driving field strength, the orientation of FE domains or PNRs toward the electric field direction brings progressive domain growth and augmentation in amplitude values in both samples. The domains are completely switched to the electric field direction at a relatively lower driving field of ≈25 V in NN ceramic (as illustrated in the comparison of amplitude and phase images in Figure S6). By comparison, the NBBLNTF ceramic exhibits a delayed polarization saturation as evidenced by a persistent trend of increasing polarization response until the driving electric field elevates to 50 V. This delayed saturation behavior facilitates a smoother polarization reversal process, effectively mitigating hysteresis losses and thereby enhancing energy storage performance. And a completely reversible process can be observed in Figure 2g_4_ as the electric field returns to 2 V. This reversible field‐induced polarization behavior is conducive to the near‐zero P_r_ value and ensures the achievement of large *W_rec_* and *η*.

**FIGURE 3:**
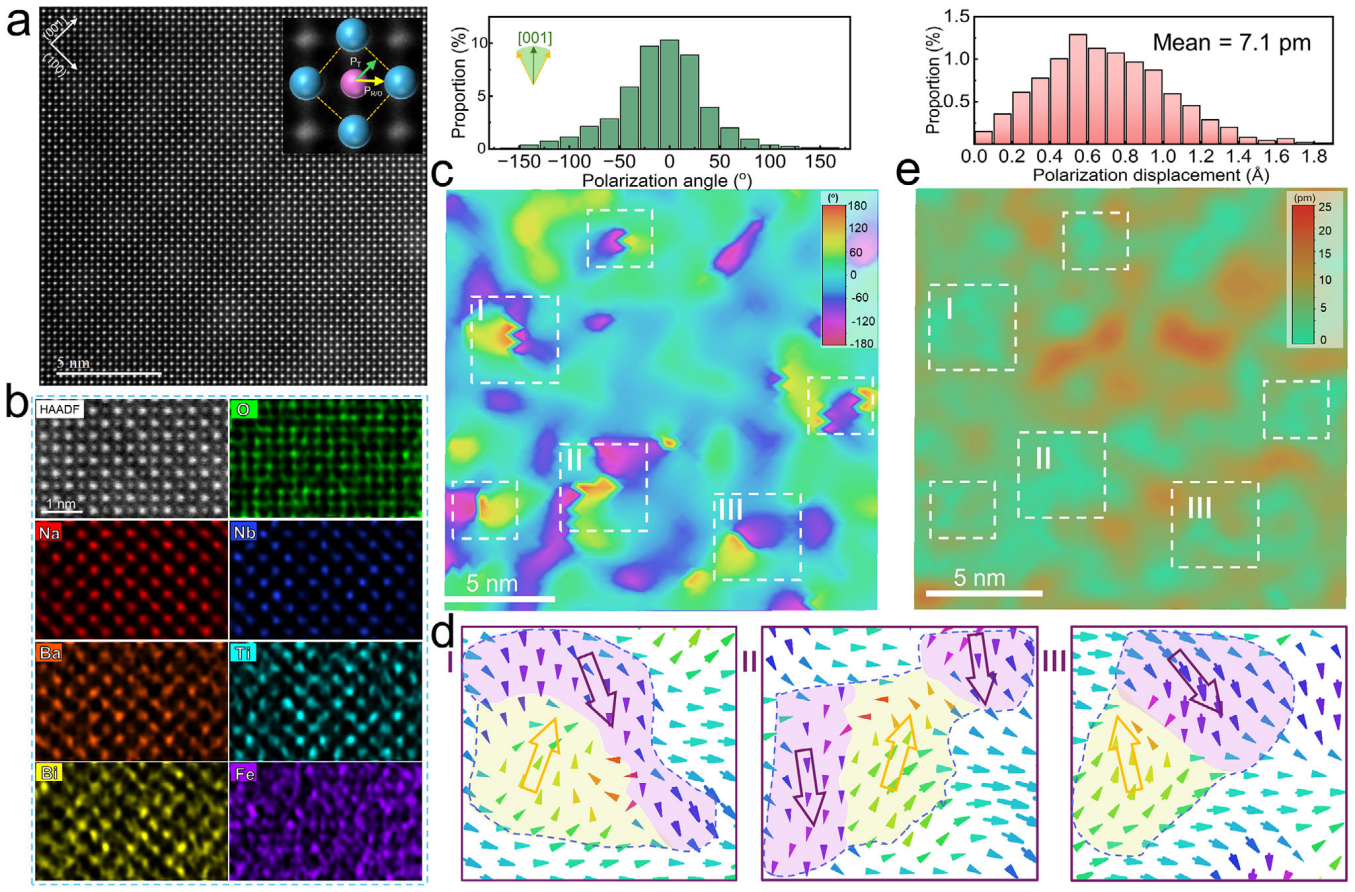
Local polarization configuration of NBBLNTF ceramic. (a) Atomic‐resolution HAADF‐STEM image of atomic structure taken with the [001]_c_ zone axis, and (b) the corresponding EDS maps for individual Na, Nb, Ba, Ti, Bi, Fe, and O elements, (c) 2D contour of polarization orientation along [001]_c_ with the statistical distributions of calculated polarization angles. (d) Magnified images with the corresponding atomic displacement vector of selected areas from (c). (e) 2D contour of polarization amplitude along [001]_c_ with the statistical distributions of calculated polarization displacement.

Aberration‐corrected scanning transmission electron microscope (STEM) and the high‐angle annular dark‐field (HAADF) STEM images were utilized to further investigate the local structural features of the NBBLNTF ceramic. Figure 3a exhibits the typical perovskite‐type atomic column arrangement along [001]_c_ zone axis, and the corresponding atomic‐resolution energy‐dispersive spectroscopy (EDS) mapping in Figure 3b reveals the uniform distributions of foreign atoms at both A‐ and B‐sites. The polarization distributions (including orientation and magnitude) can be identified by analyzing the displacement of B‐site atoms with respect to the four nearest neighboring A‐site atoms. As evidenced by the contour map and the statistical analysis in Figure 3c, NBBLNTF ceramic obtains highly disordered polarization orientation with polarization angle ranges from −180° to 180° along the [001]_c_ direction, suggesting the foreign atoms with significantly different ionic radii and ferroelectric activity effectively induce local structural distortion and hinder the formation of long‐range FE orders in the high‐entropy matrix [8]. Those multi‐symmetrical short‐range orders can reduce polarization anisotropy and enhance local random fields, contributing to the observed low‐hysteresis *P‐E* loop. Notably, the polarization orientation contour map reveals distinct local polarization configurations exhibiting pronounced angular disparities, as demarcated by the white dashed boxes in Figure 3c. The detailed distributions of polarization vectors within the specified regions (e.g., region I, II, and III) are presented in Figure 3d. Evidently, these emergent regions originate from the composition of PNRs with large polarization orientation differences (>90°), yet not perfectly antiparallel, giving rise to localized clusters that manifest distinct antipolar characteristics. Consequently, through aliovalent ions incorporation, the NBBLNTF ceramic achieves a unique combination of polarization configurations featuring quasi‐antipolar nanoclusters (QA‐NCs) embedded within a high‐entropy relaxor FE matrix.

The characteristics of the combined polarization configuration, particularly for those formed QA‐NCs, can be further investigated through the polarization amplitude distribution in Figure 3e. The QA‐NCs, outlined by white dashed boxes, exhibit significantly weak magnitudes compared to the surrounding regions that sustain strong polar fluctuations, thus breaking the FE long‐range order to form FE PNRs with sizes of 1–2 nm and resulting in a gradient polarization distribution in the NBBLNTF ceramic. Those extremely small PNRs with multiple orientations could reduce polarization anisotropy and weaken coupling between each other, thereby exhibiting high polarization responsiveness and reducing hysteresis loss [26]. And the distribution of these weak polar QA‐NCs is very analogous to the paraelectric phases in conventional RFEs, which can reduce internal stress and hinder polarization rotation under applied electric fields, thereby contributing to delayed polarization saturation [27, 28]. As the applied electric field increases, QA‐NCs would undergo a transition to polar clusters and then align along the electric field direction, thereby facilitating further enhancement of polarization intensity (as the persistently increased polarization response in PFM tests). More importantly, this transition would not cause a significant increase in hysteresis, owing to the lower transition energy barrier of QA‐NCs than that of conventional AFE nanodomains, which have a complete antiparallel arrangement. Meanwhile, these activated polar clusters can quickly recover to their initial state upon removing the electric field, further contributing to reducing hysteresis. The polarization response of NBBLNTF ceramic is compared with 0.73NN‐0.27BaTiO_3_ (NN‐0.27BT), 0.73NN‐0.27BiFeO_3_ (NN‐0.27BF), and 0.73NN‐0.27Bi_0.5_Li_0.5_TiO_3_ (NN‐0.27BLT) RFE ceramics at 20 kV/mm (Figure S7). Obviously, NBBLNTF ceramic exhibits superior energy storage potential based on a quasi‐linear *P‐E* loop with superior polarization. Therefore, the composited QA‐NCs facilitate the attainment of enhanced polarization response along with minimal hysteresis, providing a crucial structural foundation for further optimization of energy storage performance.

**FIGURE 4:**
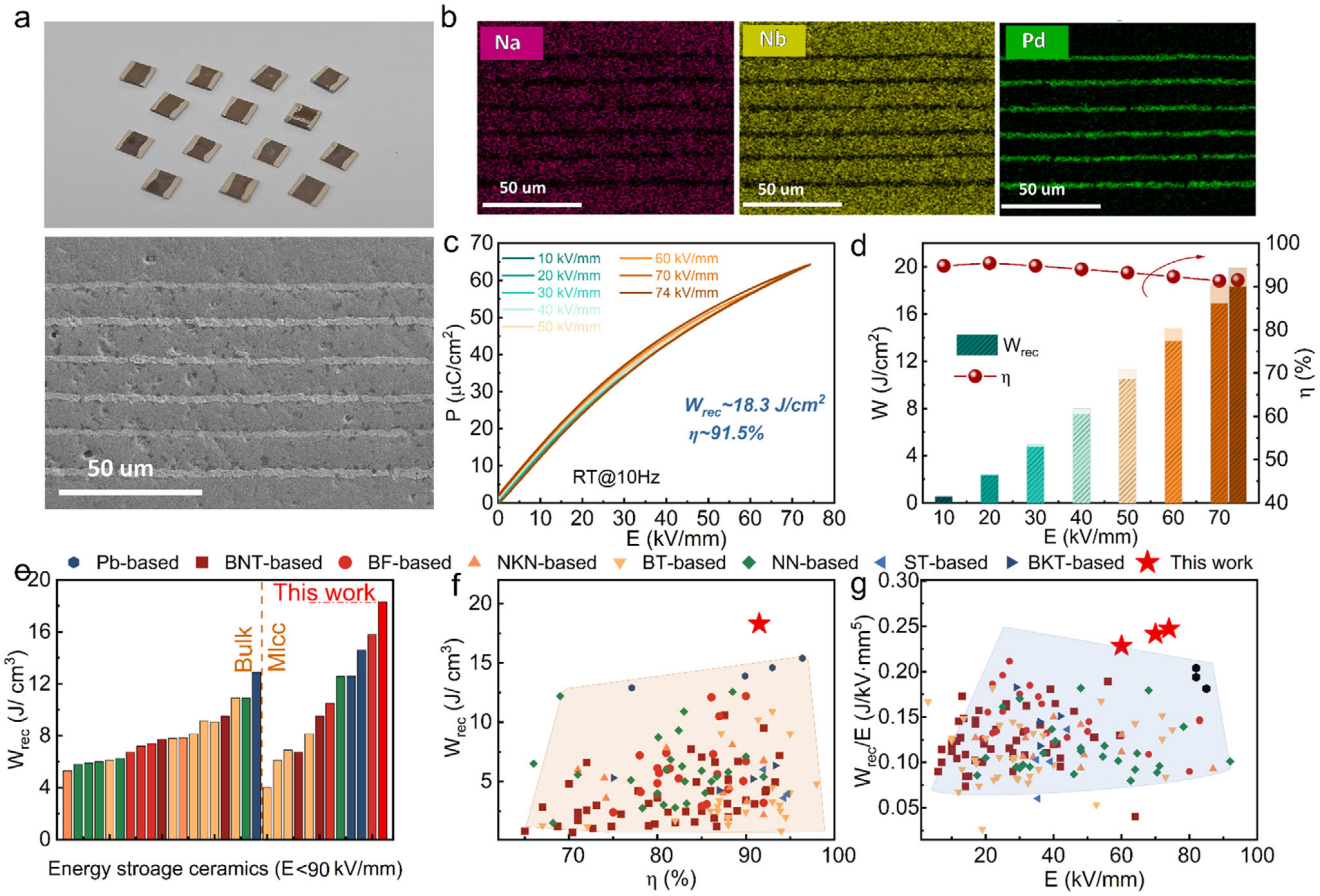
Microstructures and energy storage performance of the NBBLNTFO MLCC (a) Digital image of the MLCC and cross‐sectional SEM image with (b) corresponding element distribution. (c) *P‐E* loops up to breakdown. (d) The corresponding energy storage performance as a function of the electric field. (e) Comparisons of *W_rec_* between NBBLNTF MLCC and other reported NN‐based lead‐free bulk ceramics with electric fields < 90 kV·mm^−1^. Comparison of (f) *W_rec_* and *η*, (g) energy storage strength *W_rec_/E* of NBBLNTF MLCC with other energy storage relaxor systems.

To further verify the potential of the energy storage performance optimization strategies, the NBBLNTF prototype MLCC device was fabricated employing a traditional tape casting technique. The MLCC has five layers of dielectric, and each layer has a thickness of ≈9 µm. As shown in Figure 4a, high‐quality samples with dense dielectric layers and continuous electrode layers are obtained. The EDS results (Figure 4b; Figure S8) exhibit a clear boundary between the dielectric and electrode layer, without element diffusion. Benefiting from the reduced thickness of the dielectric layer, the breakdown field strength was further enhanced, reaching 82 kV/mm (Figure S9). The NBBLNTF MLCC exhibits a near‐linear polarization response analogous to the bulk ceramic, as shown in Figure 4c. Attributed to the enhanced ferroelectric activity and the engineering of QA‐NCs*, P_m_* increases gradually to 62.7 µC·cm^−2^ with increasing electric field, and remains a near‐zero *P_r_* at the maximum electric field. Consequently, NBBLNTF MLCC achieves an outstanding energy storage performance with a giant *W_rec_* of 18.3 J·cm^−3^ and a high *η* of 91.5% (Figure 4d). As summarized in Figure 4e,f, the ultrahigh *W_rec_* obtained in our work demonstrates a marked superiority over previously reported dielectric systems, especially with an external electric field below 90 kV·mm^−1^, showcasing the optimal overall energy storage performance among RFE ceramics, encompassing both lead‐based and lead‐free systems [6, 8, 10, 11, 12, 13, 29, 30, 31, 32, 33, 34, 35, 36, 37, 38, 39, 40, 41, 42, 43, 44, 45, 46, 47, 48, 49, 50, 51, 52, 53, 54, 55, 56, 57, 58, 59, 60, 61, 62]. Moreover, Figure 4g compares the energy storage strength *W_rec_/E* as a function of *E* between the NBBLNTF MLCC and the latest lead‐free energy storage ceramics (representative systems are tabulated in Table S1). NBBLNTF MLCC shows a superior high value of *W_rec_/E* = 0.25 J/(kV·mm^−5^), indicating its capability to achieve high energy storage density under relatively low electric fields. This remarkable characteristic holds significant implications for energy storage applications, while simultaneously validating the superiority of the proposed optimization strategy for improving energy storage performance in RFEs.

**FIGURE 5:**
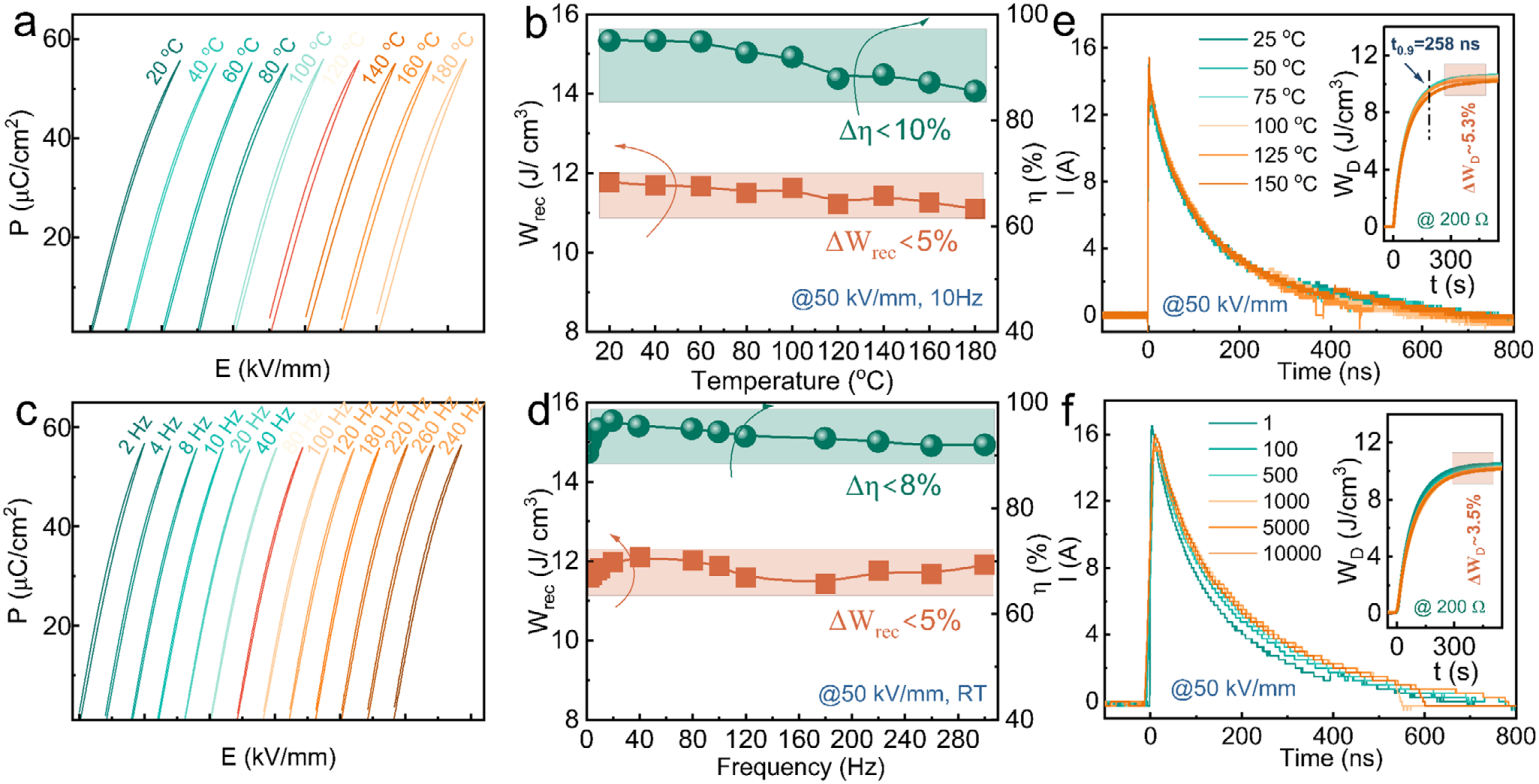
Stability and charge/discharge properties of the NBBLNTF MLCC. (a) *P‐E* loops and (b) *W_rec_* and *η* as a function of temperature at 50 kV·mm^−1^. (c) *P‐E* loops and (d) *W_rec_* and *η* as a function of frequency at 50 kV·mm^−1^. (e) Thermal stability and (f) fatigue test of (a) *P‐E* loops and (b) *W_rec_* and *η* as a function of temperature at 50 kV·mm^−1^.

From a practical application perspective, the temperature and frequency stability of the NBBLNTF MLCC was further considered to evaluate the application potential. Figure 5a exhibits the *P‐E* loops of NBBLNTF MLCC as a function of temperature under an electric field of 50 kV·mm^−1^. Slim loops with small hysteresis are obtained with increasing temperature, and the temperature‐insensitive large *W_rec_* of 11.6 ± 0.5 J·cm^−3^ (Δ*W_rec_* <5%) and a large *η* of 90 ± 4% (*η* <10%) can be maintained over a wide temperature range from 25 °C to 180 °C. The excellent thermal stability should be attributed to the temperature‐insensitive dielectric properties and phase structure, as shown in Figures S4 and S10. Regarding frequency stability, the frequency‐dependent *P‐E* loop measured from 2 to 240 Hz under 50 kV·mm^−1^ shows a significant frequency‐independent energy storage performance with a large *W_rec_* of 11.8 ± 0.2 J·cm^−3^ (Δ*W_rec_* <5%) and a large *η* of 92 ± 2% (Δ*η* ≈8%). The charge/discharge performance characteristics were also tested as an important metric for assessing the energy storage application potential. Figure 5e presents the overdamped discharge at different temperatures with a 200 Ω load resistor. The MLCC exhibits a high discharge energy density *W_D_* of 10.2 ± 0.2 J·cm^−3^ in the range of 25 °C to 100 °C and a fast discharge rate *t_0.9_* of 258 ns. Besides, the NBBLNTF MLCC obtains an excellent cycle reliability with only a slight decline in discharge energy density *W_D_* by ≈3.5% after 10^4^ charge‐discharge cycles (Figure 5f). These results reveal good charging‐discharging performance of NBBLNTF MLCC and make it a promising candidate for pulsed‐power applications.

## 3 Conclusion

In conclusion, we have successfully optimized the polarization‐field response of NN‐based lead‐free relaxor FEs by introducing QA‐NCs via a high‐entropy composition design. The resulting heterogeneous nanostructure, comprising QA‐NCs embedded within a matrix of polar nanoregions, contributes to a desirable *P‐E* loop characterized by an extremely low hysteresis, highly delayed polarization saturation, and a large *P_max_* in NBBLNTF ceramics. The fabricated NBBLNTF MLCC delivers outstanding overall energy storage performance, including an ultrahigh *W_rec_* of 18.3 J·cm^−3^, a high *η* of 91.5%, and a superior *W_rec_*/*E* of 0.25 J/(kV·mm)^−5^, along with excellent thermal (25‐180 °C) and frequency (2–240 Hz) stability. The combined merits not only underscore the promising application potential of NBBLNTF‐based devices in advanced pulsed‐power electronics but also demonstrate a feasible and effective approach to developing high‐performance energy‐storage dielectrics.

## 4 Experimental Section

### 4.1 Material Synthesis

NaNbO_3_, and Na_0.73_Ba_0.1_Bi_0.11_Li_0.06_Nb_0.73_Ti_0.22_Fe_0.05_O_3_ceramics (abbreviated as NN and NBBLNTF) were fabricated by the tape‐casting method using analytical pure raw materials of Na_2_CO_3_, Nb_2_O_5_, BaCO_3_, Bi_2_O_3_, Li_2_CO_3_, TiO_2_, and Fe_2_O_3_ (>99.0%, Sinopharm Chemical Reagent Co., Ltd). The stoichiometrically weighted powders were subjected to ball milling for 8 h and then calcined at 850 °C for 6 h. The calcined powder was ball‐milled for 12 h to obtain fine NN and NBBLNTF powders for casting. The powders were mixed with a dispersant and ethyl acetate/ethanol solvent for 24 h, and then added organic binder, plasticizer, and ball mill were added for 12 h to obtain homogenous slurries. The prepared uniform slurry was cast by a laboratory tape casting machine (CAM‐L252, KEKO, Slovenia) on a poly‐(ethylene terephthalate) (PET) substrate. The prepared green films were cut, stacked, isothermally pressured, and then cut into 1 cm × 1 cm patches, finally sintering at 1180–1220 °C for 2 h after removing the organic components. The NBBLNTF MLCC was prepared by the same green films. The 70Ag/30Pd electrode paste was screen‐printed by a printing machine using a customized mask. The printed layers were alternately stacked with blank diaphragms stacked at both ends as the protective layer, followed by isothermal pressure. After cutting and sintering, silver electrode paste was applied to both ends.

### 4.2 X‐Ray Diffraction

Phase structure was detected using a powder X‐ray diffraction (XRD) diffractometer with a Cu Kα with λ = 1.5406 Å (XRD, PANalytical X‐Pert PRO MPD, Panalytical, Netherlands).

### 4.3 Scanning Electron Microscopy

The microstructure and element distribution of ceramics and MLCC samples were examined using a scanning electron microscope (SEM EM‐30AX Plus, Coxem, Korea).

### 4.4 Piezoresponse Force Microscopy

The microscopic domain configuration under different electric fields was analyzed by piezoelectric response force microscopy (MFP‐3D, Asylum Research, USA) connected with a high voltage amplifier on polished and annealed surfaces of the ceramic samples.

### 4.5 Raman Spectra

In situ Raman spectra were acquired on well‐polished samples using a confocal microscope system (LabRam HR Evolution, HORIBA JOBIN YVON, Longjumeau Cedex, France) with a heating stage (HFS600E‐PB2, Linkam Scientific Instruments, UK).

### 4.6 Transmission Electron Microscopy

High‐angle annular dark‐field STEM (HAADF‐STEM) images were recorded on a probe‐corrected Hitachi HF5000. Specimens were prepared in a Gatan PIPS II by mechanical thinning followed by Ar⁺ ion milling.

### 4.7 Dielectric Measurements

The temperature‐, frequency‐ dependent dielectric properties, and the impedance data were measured by an LCR meter (KEYSIGHT E4990A, Keysight Technologies, USA) with a high‐temperature resistance furnace.

### 4.8 Energy Storage Properties

The ferroelectric properties were assessed based on the *P‐E* loops obtained from the ferroelectric testing platform (TF Analyzer 2000E, aixACCT Systems GmbH, Germany) integrated with a high‐temperature stage (HFS600E‐PB2, Tongguo Technology, China). The pulsed charge‐discharge current was determined using an RLC circuit (CFD‐003, Tongguo Technology, China). The energy storage properties were measured on bulk ceramic samples with a thickness of 50‐60 µm, and gold electrodes with an area of ≈0.8 mm^2^. The active area between the opposite electrodes was ≈3 mm^2^. The dielectric layers of sintered MLCCs have a thickness of ≈9 µm, and the active area between the opposite electrodes was ≈3 mm^2^.

### 4.9 Breakdown Strength

The dielectric breakdown measurements were carried out on a voltage‐withstand test device (BDJC‐50KV, Beiguangjingyi Instrument Equipment Co. Ltd., Beijing, China).

### 4.10 Leakage Current

DC leakage characteristics were evaluated using a high‐resistance measurement system (6517B, Keithley, USA) coupled with a temperature‐controlled sample stage (HCT1801, Tongguo Technology).

## Conflicts of Interest

The authors declare no conflicts of interest.

## Supporting information




**Supporting File**: advs73550‐sup‐0001‐SuppMat.docx.

## Data Availability

The data that support the findings of this study are available from the corresponding author upon reasonable request.
