# Supplementary material for: Quasi‐Antipolar Nanoclusters Driven Superior Energy Storage in High‐Entropy Relaxor Ferroelectrics
Source: Adv Sci (Weinh). 2025 Dec 30;13(14):e22801. doi: 10.1002/advs.202522801 (PMC12970253; doi:10.1002/advs.202522801)
Supplement: Supplementary file 1 — Supporting File: advs73550‐sup‐0001‐SuppMat.docx. [file ADVS-13-e22801-s001.docx]

Supporting Information

Quasi-Antipolar Nanoclusters Driven Superior Energy Storage in High-Entropy Relaxor Ferroelectrics

*Ao Tian, Zehao Li, Qingkang Jiang, Xiang Wu, Xuewen Jiang, Xin Gao, Mohamed Mahmoud, Maqbool Ur Rehman,* *Liqiang Liu, Aiwen Xie*, Tengfei Hu*, Ruzhong Zuo**

**Figure S1.** Schematic of the *P-E* loops of a) relaxor ferroelectrics, and b) antiferroelectrics.


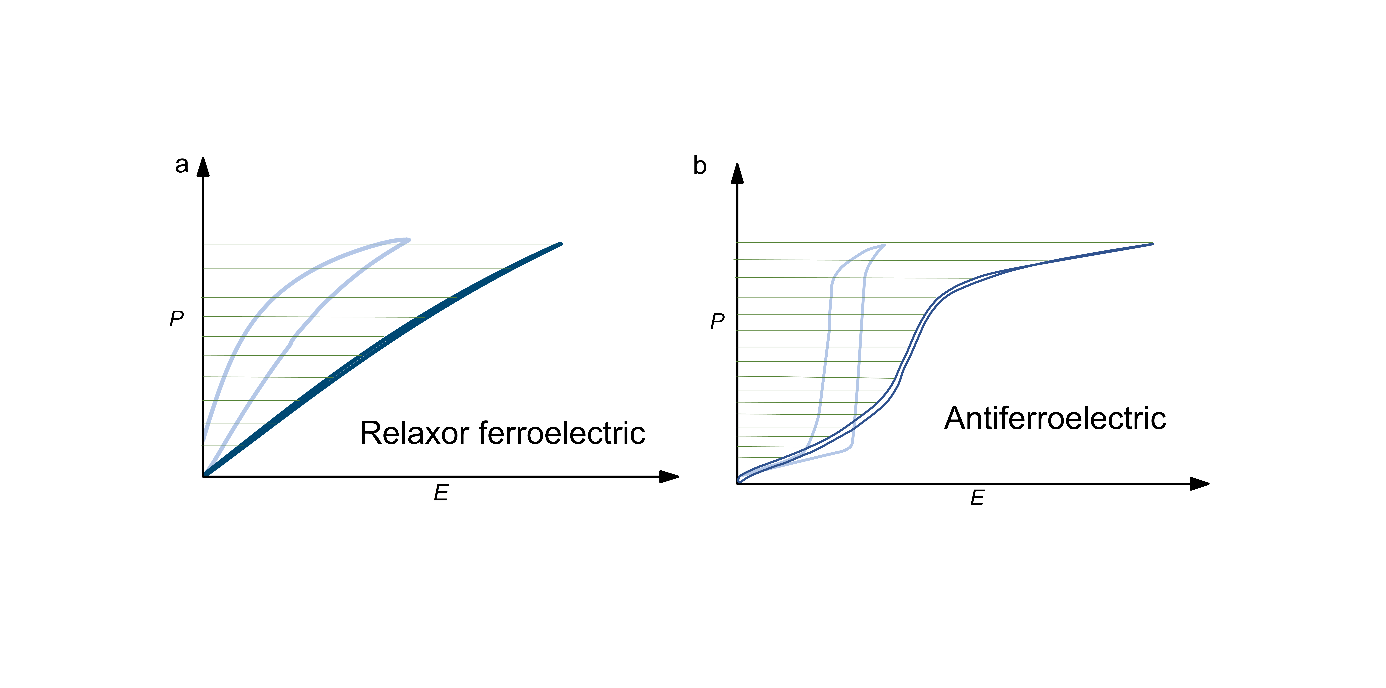


**Figure S2.** (a) XRD patterns and (b) magnified (11 3/4) and (21 3/4) peaks for NN and NBBLNTF ceramics at room temperature.


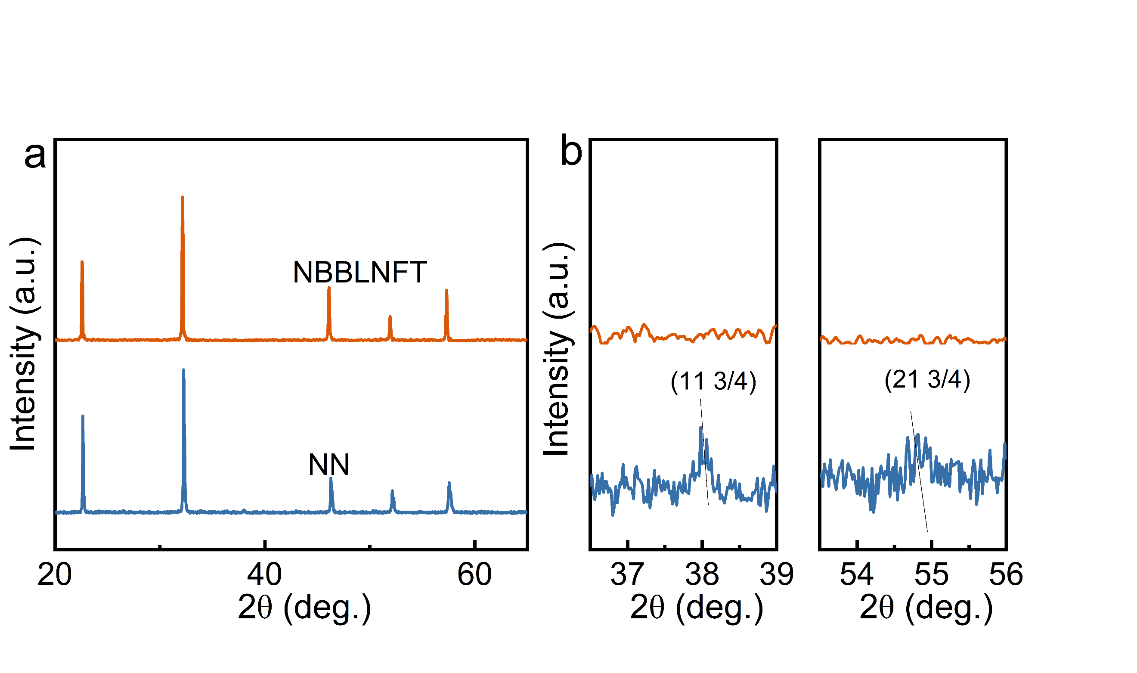


**Figure S3.** (a) Normalized dielectric constants *ε′-ε_m_* versus normalized temperature *T-T_m_*, (b) the Curie-Weiss fitting lines, and (c) *γ* and *ΔT_relax_* values of NN and NBBLNTF ceramics.


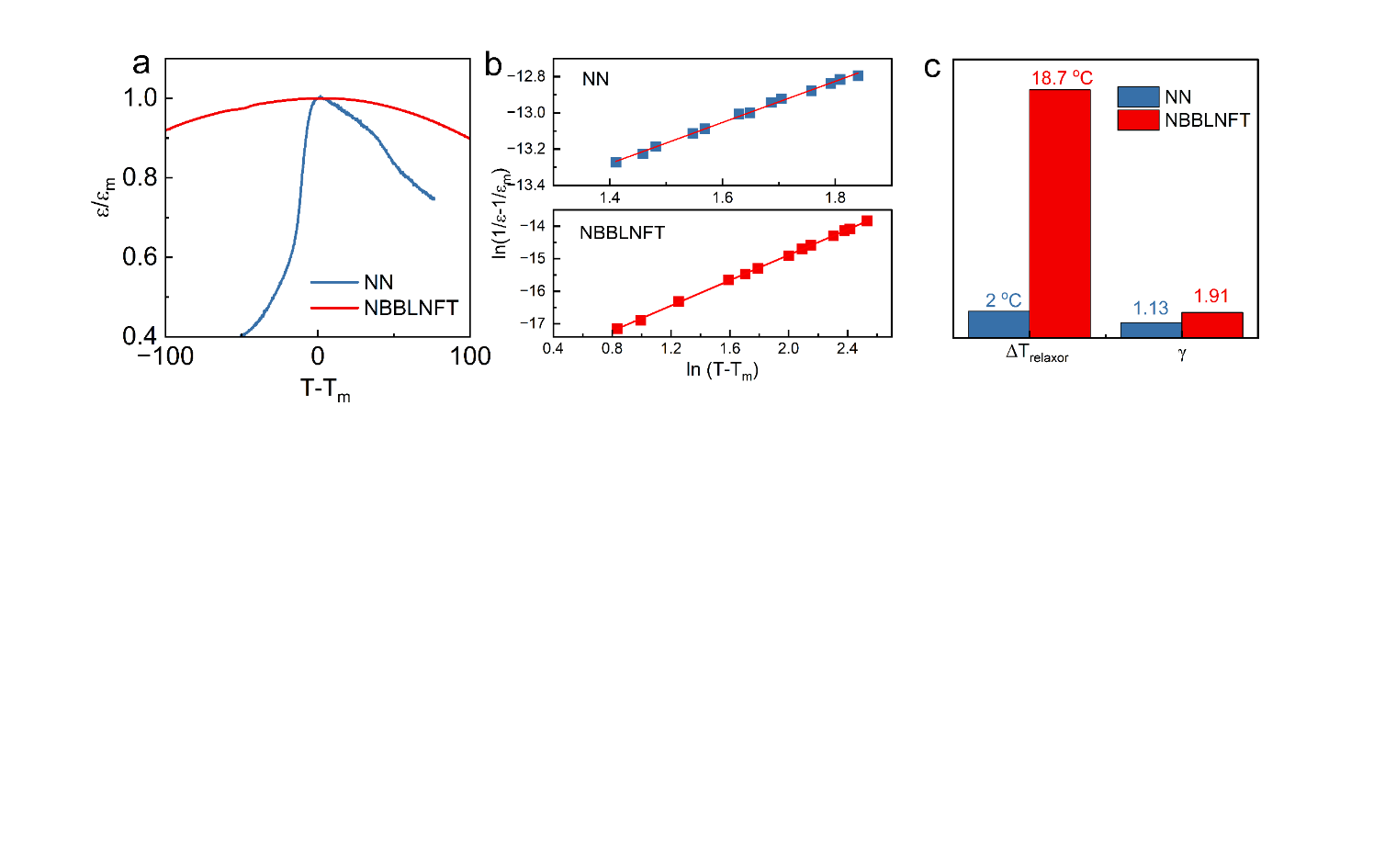


**Figure S4.** The relative variation of capacitance *ΔC/C_25_* °C as a function of temperature at 1 kHz of NBBLNTF ceramic.


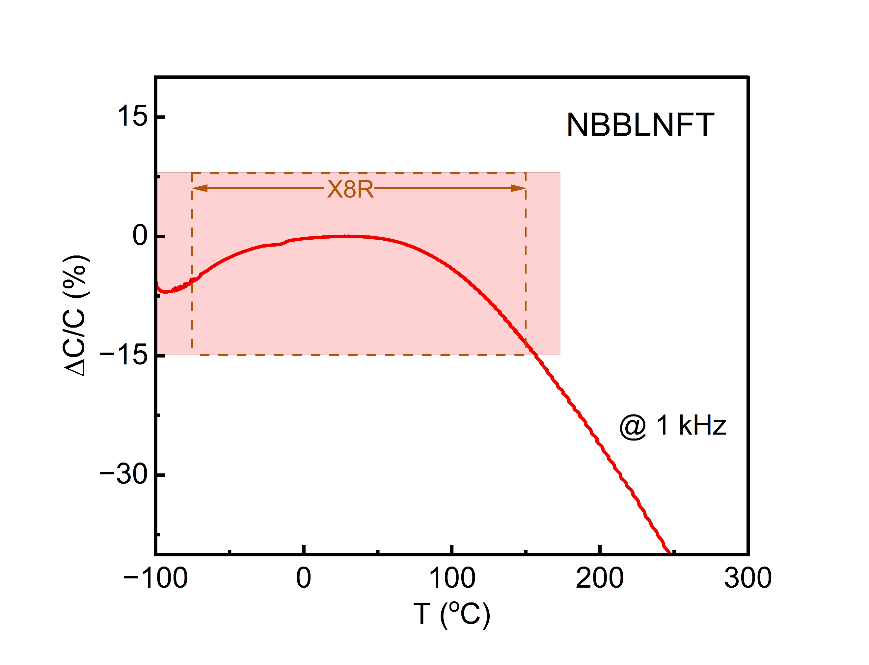


**Figure S5.** Insulation characteristics and energy-storage properties of NN and NBBLNTF ceramics. (a) Weibull distribution analysis of the breakdown strength *E_b_*. (b) Complex impedance plots measured at 500 °C and (c) the fitted resistance values. The inset of (c) shows the equivalent analog circuit. R_g_, CPE_g_, R_gb_, and CPE_gb_ represent the grain resistance, grain capacitance, grain boundary resistance, and grain boundary capacitance, respectively. (d-f) Polished and thermally-etched SEM images and grain size distribution of ceramics. (g) Frequency-dependent Z´´/Z´´_max_ at 440-520 ^o^C and (h) the E_a_ value for the NBBLNTF ceramic. (i) *I-V* curves of each component ceramic.


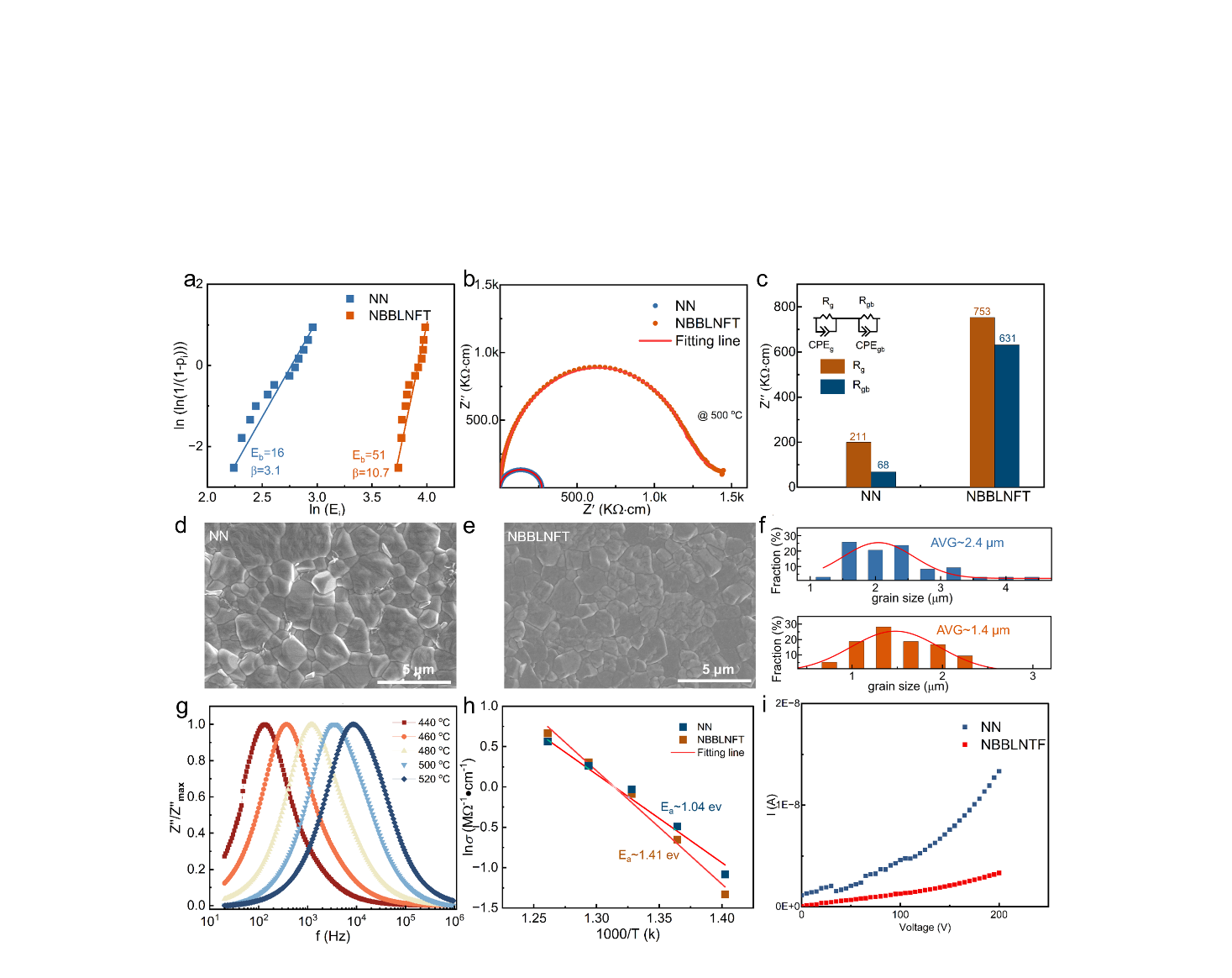


**Figure S6.** PFM amplitude and phase images at various electric fields of NN and NBBLNTF ceramics.


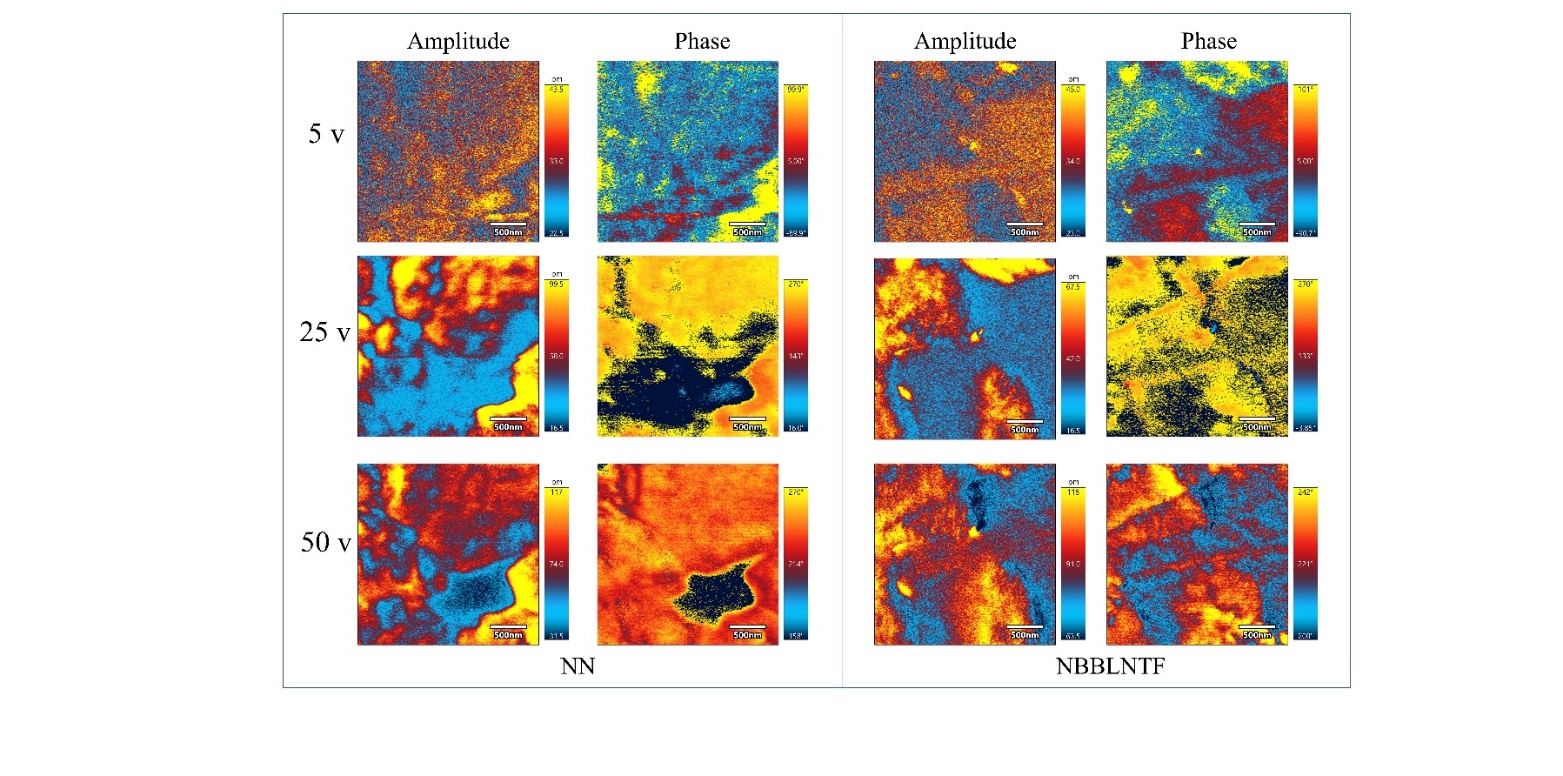


Figure S7. *P-E* loops at 20 kV/mm with a test frequency of 10 Hz and temperature-dependent dielectric properties (*ε_r_* and loss *tanδ*) for NN-0.27BT, NN-0.27BF, and NN-0.27BLT ceramics.


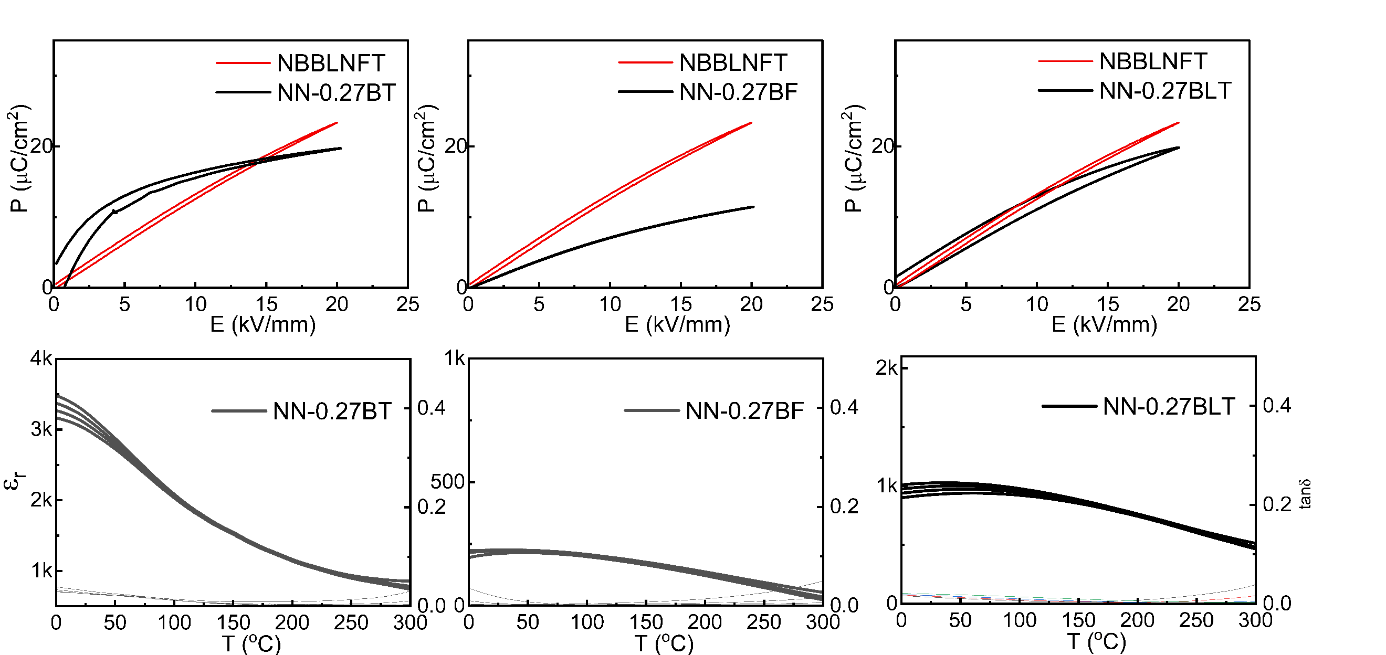


**Figure S8**. The cross-sectional SEM image and the corresponding element distribution of the NBBLNTFO MLCC.


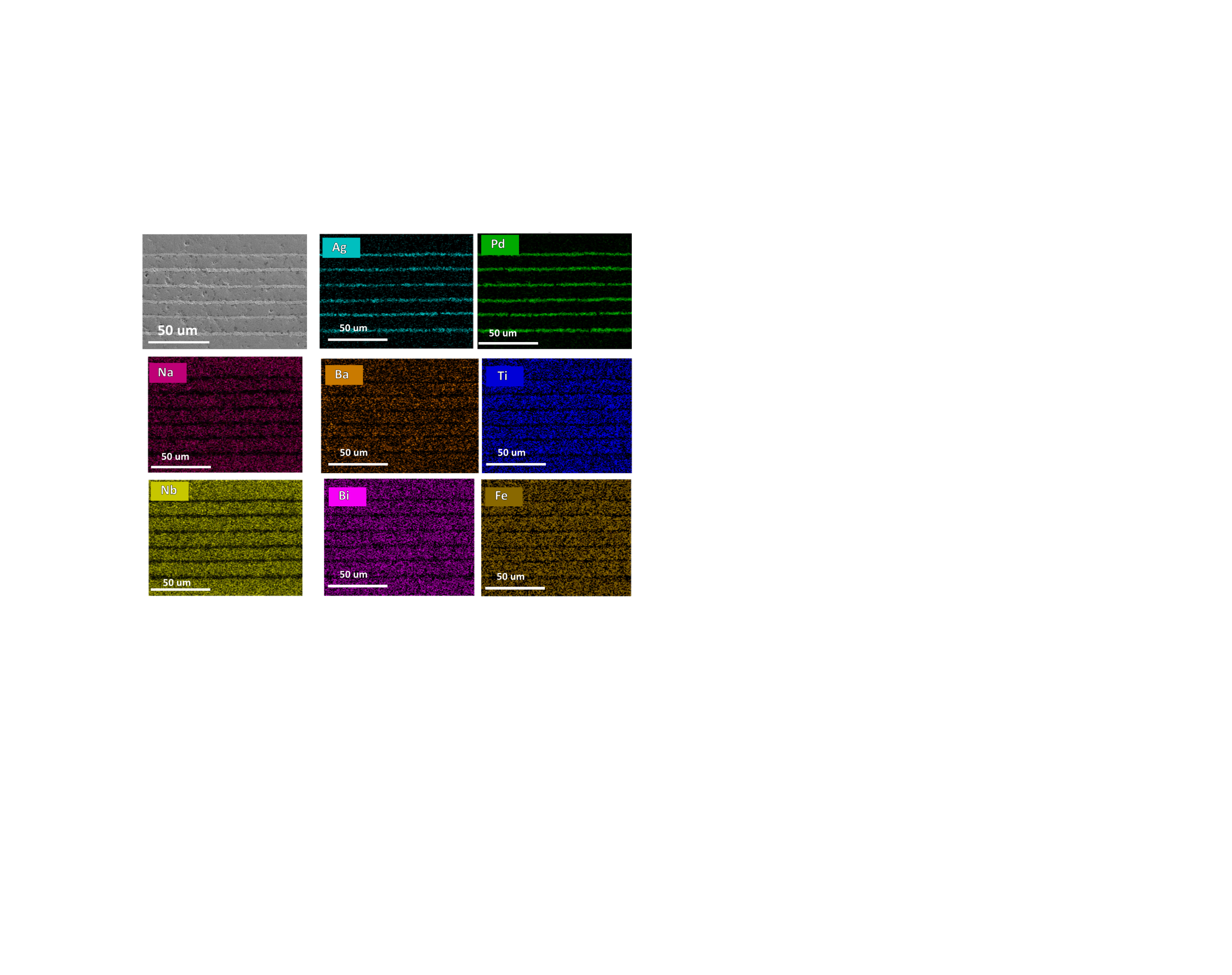


**Figure S9**. Weibull distribution analysis of the breakdown strength E_b_ of NBBLNTF MLCC.


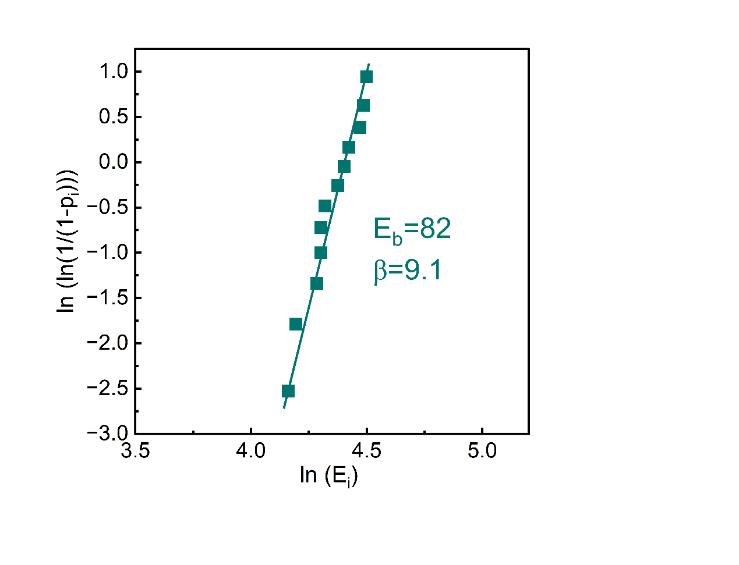


**Figure S10.** Temperature-dependent (a) XRD patterns and (b) Raman spectrum of NBBLNTF ceramic. (c) The variation of the wavenumber of υ1 and υ6 Raman modes as a function of temperature.


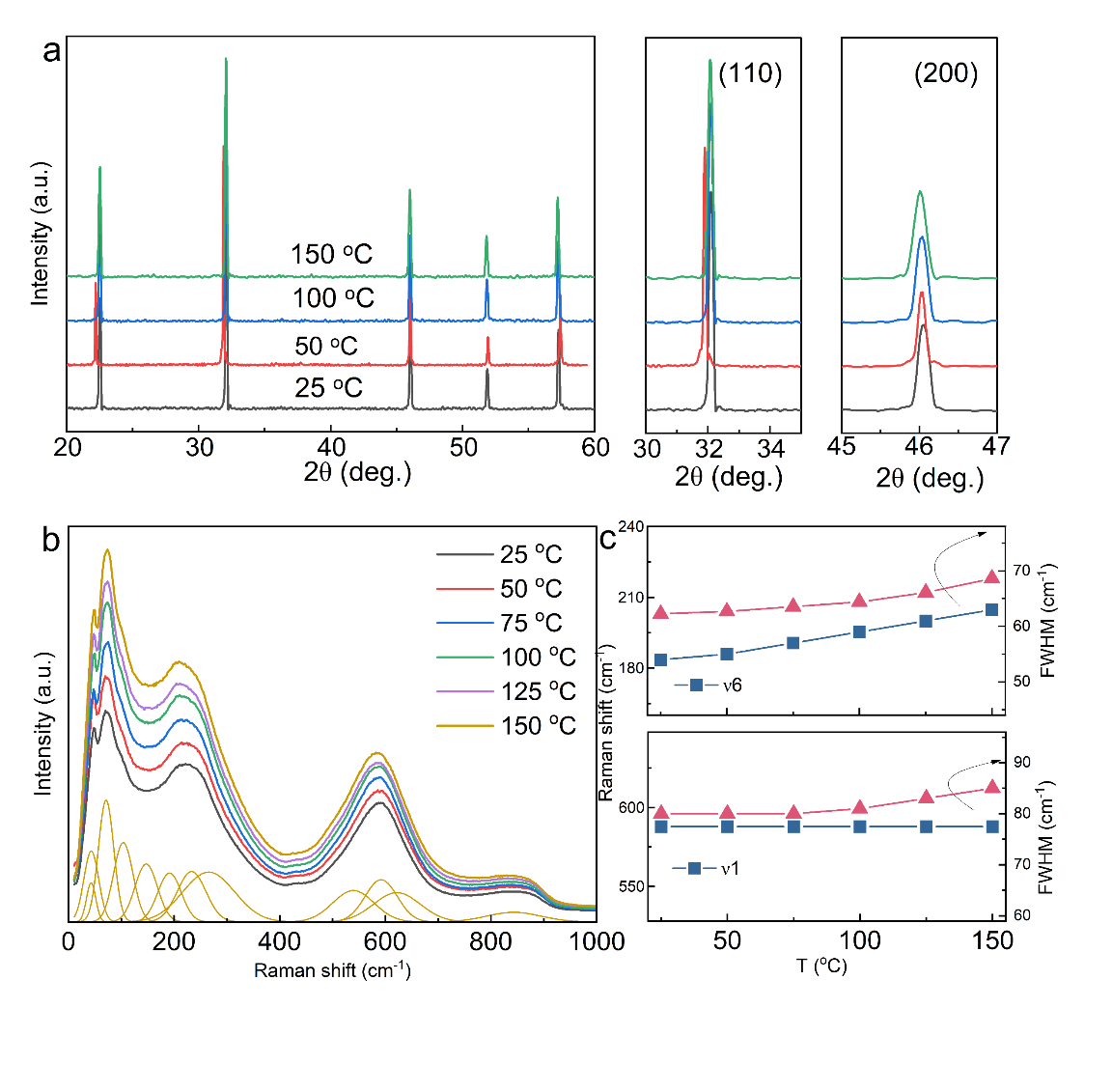


Table S1. Energy-storage performance of representative systems.

| **Composite** | **Thickness (μm)** | **E (kV/mm)** | **W_rec_ (J/cm^2^)** | **W/E (J·(kV/mm)^5^)** | **η (%)** | **Ref.** |
| --- | --- | --- | --- | --- | --- | --- |
| BiFeO_3_-0.8 (Ba_0.2_Sr_0.2_Ca_0.2_Bi_0.2_Na_0.2_)TiO_3_ | 12 | 83 | 12.1 | 0.15 | 86.1 | [1] |
| 0.67BiFeO_3_-0.33BaTiO_3_-xLaAlO_3_ | / | 27 | 5.71 | 0.21 | 80.19 | [2] |
| 0.6Bi_0.9_La_0.1_FeO_3_-0.3Ba_0.7_Sr_0.3_TiO_3_-0.1Na_0.5_Nb_0.85_Ta_0.15_O_3_ | 50 | 72 | 15.9 | 0.22 | 87.7 | [3] |
| 1/3BaTiO_3_-1/3(Bi_0.5_Na_0.5_)TiO_3_-1/3NaNbO_3_ | 60 | 54 | 10.59 | 0.19 | 87.6 | [4] |
| 0.85BaTiO_3_-0.15(Bi_0.5_Na_0.5_)(Zn_1/3_Nb_2/3_)O_3_ | 70 | 58 | 11.6 | 0.2 | 96.1 | [5] |
| Ba_0.82_Bi_0.12_TiO_3_ | 100 | 70 | 10.1 | 0.14 | 90 | [6] |
| BiFeO_3_-0.65SrTiO_3_ | 36 | 75 | 8.4 | 0.11 | 90 | [7] |
| (Bi_0.5_Na_0.5_)_0.65_Sr_0.35_TiO_3_-0.10Bi(Mg_0.5_Zr_0.5_)O_3_ | 100 | 52.2 | 8.46 | 0.16 | 85.9 | [8] |
| (Bi_0.5_Na_0.5_)TiO_3_-0.30SrTiO_3_-0.2(Sc_0.5_Ta_0.5_)^4+^ | 10 | 59 | 12.2 | 0.20 | 85.9 | [9] |
| (0.94Na_0.5_Bi_0.5_TiO_3_-0.06BaTiO_3_)-0.38Ca_0.7_La_0.2_TiO_3_ | 120 | 64 | 15.1 | 0.23 | 82.4 | [10] |
| Bi_0.25_Na_0.25_Ba_0.5_Ti_0.92_Hf_0.08_O_3_ | 50 | 80 | 16.21 | 0.2 | 90.5 | [11] |
| (0.75Na_0.5_Bi_0.5_TiO_3_-0.25BaTiO_3_)-0.12NaNbO_3_ | 70 | 72 | 15.2 | 0.21 | 91 | [12] |
| (0.94BNT-0.06BaTiO_3_)-0.3Sr_0.7_Nd_0.2_TiO_3_ | 70-150 | 54 | 8.08 | 0.15 | 92.1 | [13] |
| Bi_0.5_Na_0.5_TiO_3_-0.15AgNb_0.5_Ta_0.5_O_3_ | 120 | 51 | 6.6 | 0.13 | 72 | [14] |
| K_0.5_Na_0.5_NbO_3_-0.09(Sr_0.7_La_0.2_)(Mg_1/3_Ta_2/3_)O_3_ | 150 | 40 | 6 | 0.15 | 71 | [15] |
| (0.75Bi_0.5_K_0.5_TiO_3_-0.25BiFeO_3_)-0.5CaTiO_3_ | / | 42 | 6.4 | 0.15 | 93.32 | [16] |
| (Na_0.5_K_0.5_)NbO_3_-0.1Bi(Zn_2/3_(Nb_0.5_Ta_0.5_)_1/3_)O_3_ | 50 | 31 | 3.92 | 0.13 | 91 | [17] |
| NaNbO_3_-0.12Bi(Mg_2/3_Nb_1/3_)O_3_-0.05CaZrO_3_ | 80-100 | 64 | 5.9 | 0.1 | 85 | [18] |
| (Na_0.91_Bi_0.09_)(Nb_0.94_Mg_0.06_)O_3_ | / | 78.3 | 10.9 | 0.14 | 83 | [19] |
| 0.91Sr_0.6_(Na_0.5_Bi_0.5_)_0.4_TiO_3_-0.09BaBi_2_Nb_2_O_9_ | / | 35 | 3.6 | 0.10 | 94.3 | [20] |
| Na_0.73_Ba_0.1_Bi_0.11_Li0.06Nb_0.73_Ti_0.22_Fe_0.05_O_3_ | 9 | 74 | 18.3 | 0.25 | 91.5 | This work |

[1] H. Li, X. Li, Y. Du, X. Chen, H. Qin, Y. Tabak, A. Evcin, F. Hussain, K. Song, H. Zhou, J. Zhao, D. Wang, *Chem. Eng. J.* **2024**, *499*, 156112.

[2] J. Zhang, Y. Pu, Y. Hao, Y. Yang, L. Zhang, B. Wang, Q. Pan, *J. Energy Storage.* **2025**, *105*, 114786.

[3] T. Cui, J. Zhang, J. Guo, X. Li, S. Guo, Y. Huan, J. Wang, S.-T. Zhang, Y. Wang, *Acta Mater.* **2022**, *240*, 118286.

[4] L. Chen, N. Wang, Z. Zhang, H. Yu, J. Wu, S. Deng, H. Liu, H. Qi, J. Chen, *Adv. Mater.* **2022**, *34*, 2205787.

[5] L. Chen, T. Hu, X. Shi, H. Yu, H. Zhang, J. Wu, Z. Fu, H. Qi, J. Chen, *Adv. Mater.* **2024**, *36*, 2313285.

[6] Z. Sun, J. Zhang, H. Luo, Y. Yao, N. Wang, L. Chen, T. Li, C. Hu, H. Qi, S. Deng, L. C. Gallington, Y. Zhang, J. C. Neuefeind, H. Liu, J. Chen, *J. Am. Chem. Soc.* **2023**, *145*, 6194.

[7] F. Yan, H. Bai, G. Ge, J. Lin, C. Shi, K. Zhu, B. Shen, J. Zhai, S. Zhang, *Small* **2022**, *18*, 2106515.

[8] X. Zhu, Y. Gao, P. Shi, R. Kang, F. Kang, W. Qiao, J. Zhao, Z. Wang, Y. Yuan, X. Lou, *Nano Energy* **2022**, *98*, 107276.

[9] B. He, T. Ochirkhuyag, W. Feng, M. Liu, S. Liu, Z. Bao, C. Hu, Y. Zhong, D. Odkhuu, *J. Mater. Chem. A* **2023**, *11*, 14169.

[10] W. Cao, R. Lin, X. Hou, L. Li, F. Li, D. Bo, B. Ge, D. Song, J. Zhang, Z. Cheng, C. Wang, *Adv. Funct. Mater.* **2023**, *33*, 2301027.

[11] H. Luo, Z. Sun, J. Zhang, H. Xie, Y. Yao, T. Li, C. Lou, H. Zheng, N. Wang, S. Deng, L.-F. Zhu, J. Liu, J. C. Neuefeind, M. G. Tucker, M. Tang, H. Liu, J. Chen, *J. Am. Chem. Soc.* **2024**, *146*, 460.

[12] H. Liu, Z. Sun, J. Zhang, H. Luo, Y. Yao, X. Wang, H. Qi, S. Deng, J. Liu, L. C. Gallington, Y. Zhang, J. C. Neuefeind, J. Chen, *J. Am. Chem. Soc.* **2023**, *145*, 19396.

[13] C. Long, Z. Su, A. Xu, H. Huang, L. Liu, L. Gu, W. Ren, H. Wu, X. Ding, *Nano Energy* **2024**, *124*, 109493.

[14] Z. Che, L. Ma, G. Luo, C. Xu, Z. Cen, Q. Feng, X. Chen, K. Ren, N. Luo, *Nano Energy* **2022**, *100*, 107484.

[15] X. Duan, J. Wang, Q. Chai, P. Ren, H. Du, L. Jin, F. Lai, Z. Peng, X. Chao, J. Lu, Q. Guo, B. Xie, *J. Power Sources* **2025**, *649*, 237453.

[16] L. Zheng, Z. Niu, P. Zheng, K. Zhang, C. Luo, J. Zhang, N. Wang, W. Bai, Y. Zhang, *Mater. Today Energy* **2022**, *28*, 101078.

[17] L. Hou, C. Li, X. Wang, X. Wang, T. Wang, Y. Huan, *J. Adv. Dielect.* **2023**, *13*, 2242001.

[18] L. Zhang, Z. Chen, G. Luo, F. Ai, N. Luo, *J. Eur. Ceram. Soc.* **2023**, *43*, 6077.

[19] J. Jiang, X. Meng, L. Li, J. Zhang, S. Guo, J. Wang, X. Hao, H. Zhu, S.-T. Zhang, *Chem. Eng. J.* **2021**, *422*, 130130.

[20] C. Wu, Y. Pu, X. Lu, Y. Ning, B. Wang, L. Zhang, Z. Chen, Y. Yang, *J. Power Sources* **2024**, *604*, 234475.
